# Methods of Diagnosis in Gastric Cancer

**Published:** 1913-06

**Authors:** J. M. Fortescue-Brickdale

**Affiliations:** Assistant Physician to the Bristol Royal Infirmary and Clinical Lecturer in the University of Bristol.


### METHODS OF DIAGNOSIS IN GASTRIC CANCER.

BY

J. M. Fortescue-Brickdale, M.A., M.D. Oxon.,

*Assistant Physician to the Bristol Royal Infirmary and Clinical Lecturer in the University of Bristol.*

---

The importance of early diagnosis in cases of cancer of the stomach has given rise to a large amount of clinical and laboratory work, the object of which has been to produce some test or sign so characteristic of the early stages of the disease as to make it possible for a physician to recommend and a surgeon to employ operative treatment at a time when it is likely to be of positive advantage to the patient. I propose to consider the value of the various tests which have been devised, and in order to do so it will be convenient to classify them into three main groups. In the first group I would place those tests which are applied to the contents of the stomach; in the second, those applied to the blood; and in the third, those applied to the urine.

### I. Tests applied to the Gastric Contents.

These may be subdivided into three smaller groups—

1. Those which show stasis of the stomach contents.

These are three in number, namely the absence of hydrochloric acid,[1] the presence of lactic and other organic acids, and the presence of the so-called Opler-Boas bacilli.

2. In the second group are those tests which show the presence of ulceration, namely the various methods for demonstrating minute quantities of blood in the gastric contents or fæces [2] and the tests for serum albumen in the stomach washings (Salomon's test). [3]

These tests have been introduced for some years, and their value and limitations are now well recognised. [4] The demontration of stasis or of an ulcerated surface is a valuable additional item in diagnosis, but their presence or absence are of no preponderating or conclusive significance. They may not even be early occurrences in the course of a gastric cancer, so that no special reliance can be placed on their presence or absence as indications for or against operation.

3. The third group consists of a number of tests for alterations in the gastric contents, due to the special influence of carcinomatous growths.

(*a*) *Gluzinski's test.* [5]—When an ulcer heals or becomes carcinomatous the HCl fails and a mucous gastritis supervenes. Gluzinski considers that the former occurrence is so rare as to be negligible. The stomach contents have to be removed three times on the same day and tested for free HCl and total acidity. The published cases do not seem very convincing.

(*b*) A more simple test is that for dissolved albumins. [6] Wolff states that in simple achylia only a small amount of the albuminous matter in a test meal is dissolved, whereas in carcinoma a high percentage of dissolved albumin is found. The reagent used is phosphotungstic acid, prepared according to a formula devised by Wolff, and is added to a series of dilutions of the filtered gastric contents. If much albumen is present high dilutions will, of course, show a cloudy precipitate and vice versa. The only difficulty about the practical value of this test is that, though the findings are clear enough in undoubted cases of cancer, the border-line cases are not so easily decided. Medium degrees of dissolved albumin may or may not indicate cancer. The finding of very small amounts is no doubt very much against the diagnosis.

(*c*) It has been suggested that the reason why there is often little free HCl in the gastric contents in cases of cancer is that the albumens are split up into amino-acids, which combine with more HCl than the original large protein molecule.

However this may be, it seems clear that under certain conditions increased splitting of proteins does occur in cancer of the stomach, and this is the basis of the well-known tryptophane test.[7] This test is apt to be interfered with by the presence of blood or bile in the stomach. Blood in dilution of 1 in 500 may upset the test, and in greater amounts certainly will do so. Regurgitation of trypsin from the duodenum and increased protein-splitting by bacterial action may also vitiate this test. Moreover, it sometimes gives a negative result in early cases, and may give a positive result in other conditions, such as ulcer and simple achylia. If a number of tests are made, however, a series of negatives may be taken as very much against cancer. Schryver and Singer,[8] in the autumn of last year, published some observations showing that a ferment capable of splitting peptone occurred in numerous conditions of severe gastric disease, that it was not characteristic of cancer, and was most commonly associated with dilatation and achylia.

(*d*) The toxicity of the gastric juice to guinea-pigs has also been employed as an early test for gastric cancer. Liveriato[9] injected it subdurally, and found that whereas 1 c.c. from normal stomachs produced no symptoms, 0.1 c.c. from cancer cases was sometimes fatal. Anaphylaxis was produced by the maximum harmless dose of 0.05 c.c., which never occurred with normal gastric juice. The guinea-pigs were prepared by an injection of extract of carcinoma, and then given subdurally a small amount of filtered gastric juice, obtained three-quarters of an hour after an ordinary Ewald test meal. A marked symptom in the anaphylactic shock produced was a fall of temperature. Control experiments with normal gastric juice and with non-sensitised animals were negative. Similar reactions have been obtained by other workers for blood serum, and not only in cancer but in syphilis, tuberculosis and other diseases. The reaction in any case appears to be against the foreign cells and not against the specific cause of cancer, whatever that may be. Embryonic cells, other neoplasms, and apparently normal sera occasionally give anaphylactic reactions in the sensitised guinea-pigs.

(*e*) Attempts have also been made to obtain specific precipitins from the gastric contents in cancer. Alfredo[10] prepared a rabbit serum by injecting cancer extracts, and this was tested against gastric juice *in vitro*. Cancer juices were found to precipitate the cancer serum, but apparently gastric juice in other conditions, *e.g.* ulcer, may produce a similar result.

In reviewing the methods described in this group, it will be noted that they are far more specific in character than those of the first two groups. Gluzinski's test is in a way intermediate between this group and the others, as it represents a *result* of the malignant growth on the secretory processes in the stomach, which, though not specific, is somewhat characteristic. Many possible sources of error surround the tryptophane test, and in early cases it is not infrequently negative. These tests may therefore be considered not sufficiently biological in character, while those depending on the production of anaphylactic shock are, if anything, *too* biological, and do not rest on a sufficient knowledge of the underlying principles of cellular physiology.

### II. Tests applied to the Blood.

Passing on to the second main group of tests, two main subgroups will have to be considered. The first sub-group consists of tests applied to the corpuscular part of the blood, and need not detain us for long. Two diagnostic points have been suggested as of value, namely the failure of digestion leucocytosis and the decrease in the hæmoglobin. The former is now recognised as unreliable, and the latter is not shown to occur sufficiently early to be of practical value.

We can therefore go on at once to consider the tests applied to the blood serum, and it is to be noted that the general criticism levelled against all these tests is that they are most likely indications that the originally local disease has become general, and therefore that the time at which diagnosis is essentially important has gone by. In some cases this may indeed be true, but it does not altogether follow that because alterations have been produced in the blood serum therefore actual metastasis must have taken place. Nor do we know,

I take it, how much "generalisation" is necessary to prevent successful surgical treatment of a primary growth. These tests, therefore, must be judged on their merits, with regard firstly to their clinical accuracy and secondly to their practical simplicity.

1. Macalister and Ross[11] have stated that the blood of cancer patients contains a substance or substances which excite pseudopodial activity in normal leucocytes, a reaction also observed with certain alkaloids. How early in a case of cancer this reaction may be expected to occur does not appear from their original communication; and although they did not obtain any positive results in healthy persons or those suffering from diseases other than cancer, it is quite possible, as they themselves admit, that the phenomenon they describe is not really specific at all.

2. Two tests which may next be described seem to be connected with each other in some way, though not altogether running on parallel lines. The first of these is the reaction described by Brieger, Trebing and Marcus, and sometimes called Brieger's cachexia reaction. Normal blood serum contains a substance which is capable of inhibiting the proteolytic action of trypsin. The serum of guinea-pigs immunised to trypsin shows this property in a much higher degree. In certain diseases also the antitryptic power of the serum varies considerably. The test is usually carried out by dropping varying amounts of trypsin solution mixed with a constant amount of blood serum on to Petri dishes filled with a solid medium made from ox serum and incubating them. Where the trypsin has acted on the medium small depressions are found, and the antitryptic action of the serum to be tested is measured by the strength of dilution of trypsin it can inhibit. Normal blood serum apparently inhibits trypsin solution in 1 to 4 dilution. The antitryptic power is much decreased during digestion and often in diabetes; it has also been found decreased in chronic Graves's disease, primary untreated syphilis, and in catarrhal jaundice. On the other hand, in pneumonia, it is increased up to the crisis, and then falls to normal as the physical signs disappear, and in lymphatic leukæmia and various blood diseases it is also increased, but not always to any great extent. In a large number of cases of carcinoma the antitryptic power of the serum is considerably increased, thus the proportion may be 1:10 or 1:8 instead of the normal 1:4. Other cases of cancer give only a slight increase, such as might be found in normal persons. Now in some patients showing an increase in the antitryptic action of the serum the increase disappears after trypsin has been given by the mouth, whereas in others a still further increase occurs and is maintained. This latter reaction is taken to indicate a rather worse prognosis. Brieger considers that the increased antitryptic power of the serum is a measure of the cachexia induced by the disease. It is not specific for cancer, as it occurs in other chronic cachexias, but it may be of value in prognosis, and a negative result of the test is against the presence of cancer.

3. The next test is that described by Shaw-Mackenzie,[13] namely the increased power of activating the fat-splitting pancreatic ferment which is possessed by the serum of carcinomatous patients. According to his observations, human and animal serum itself has no lipoclastic or fat-splitting action, but is capable of activating pancreatic lipase. This activating power is much increased in carcinoma and in certain other diseases, such as diabetes and possibly tuberculosis. This reaction which, unlike the antitryptic action, has an accelerating and not an inhibiting effect, is also unlike it in that it persists after recovery or removal of the growth, whereas the antitryptic power sinks to normal under these conditions.

The lipase, according to Rosenheim's experiments, consists of two parts, which may be separated by filtering a glycerine extract of pancreas. The solid residue is inactive and the filtrate almost inactive, but mixed together they are active. The solid residue is thermolabile, and may be activated not only by the thermostable filtrate, but by normal blood serum and to a still greater extent by carcinomatous blood serum. The activity is measured by titration of the fatty acids produced. Shaw-Mackenzie believes that this reaction, together with the antitryptic, are strong evidence of the presence of cancer, while their absence excludes it. It is, however, not yet clear whether these reactions are constantly present in the very early stages of the growth.

4. Freund and Kaminer[14] noted that normal serum contained a substance which dissolves cancer cells, whereas serum from patients with carcinoma failed to do so. This reaction, however, is not constant, and there are considerable difficulties in obtaining a suitable emulsion of cancer cells. Monakow found that sera of all cancer cases showed a destructive action on cancer cells in at least one-fifth of the cases. Other diseases give the reaction in at least 15 per cent. of the cases, and in three early cases of cancer it was only positive in one (Kraus, Graff and Ranzi).

5. Cancer serum has also been found in certain cases to dissolve normal red corpuscles, though not those of the patient.[15] This reaction is again not constant, and has been calculated to be present in only about 40 per cent. of the cancer serums. A similar reaction, though apparently to a lesser extent, has been shown to occur in tuberculosis and in other diseases. The hæmolytic action is destroyed by heating to 50 deg., but after heating the activity is restored by admixture of fresh normal serum. The reaction, therefore, appears to be due to a substance belonging to the class of bodies like the solid residue of pancreatic extract above described, that is to the amboceptors or immune bodies. It has been named isohæmolysis. A modification of this test, in which the hæmolysis is made to take place subcutaneously by injecting a suspension of normal corpuscles under the skin of the cancerous patient, was introduced by Elsberg and others in 1908. Various changes took place within a few hours at the site of injection, which were said to be characteristic of cancerous hæmolysis. The originators claimed 77 per cent. of positive results in cancer, but Risley, who repeated their experiments, only got 33.5 per cent. of positive results in cancer, while only 75 per cent. of non-malignant cases were negative. Moss investigated the natural hæmolysins, and found that though not present so generally as the agglutinins, they seemed to be governed by similar laws.

Considerable variation in agglutinative power of normal serum and in responsive reaction to agglutinins in red corpuscles occur in different individuals ; some sera contain no agglutinins, and some corpuscles never agglutinate. Other sera will only agglutinate certain corpuscles, and again, some corpuscles can only be agglutinated by certain sera. Gorham and Lissen have shown that these conditions also apply to a great extent to hæmolysis. By using Elsberg's method they obtained 60 per cent. positive results in cancer and 88 per cent. negative results in non-cancerous cases. The patients were tested with four different groups of corpuscles. They consider that the hæmolytic test is certainly not specific, and that the results obtained by test-tube experiments are quite different from those obtained by the subcutaneous method. Considerable importance attaches to the sort of corpuscles used, owing to the varying capacity for agglutination in the corpuscles of different individuals. They find positive reactions more significant than negative, but note that at present there is no evidence of the value of this test in doubtful or border-line cases.

6. The complement deviation method has also been extensively tried in cancer with somewhat confusing results. A large number of synthetic antigens have been tested, as well as various antigens prepared from malignant growths. The synthetic lipoids give equally strong reactions with malignant and non-malignant cases, and the various methods for preparing antigens from tumours or pancreatic extracts give bodies which are neither stable nor specific. In particular it has been found impossible to differentiate syphilis from cancer by this test.

7. The remaining test in this group is that known as the meiostagmin reaction.[17] Traube showed that the addition of a toxin to an anti-toxin caused a diminution of surface tension, and consequently that the size of a drop of the liquid was diminished and a given volume of the fluid would yield an increased number of drops. The instrument he devised for measuring the drops is called a stalagmometer, and consists of a finely-graduated pipette with a central bulb, holding about 8 c.c. The lower end forms a capillary tube, the extremity of which is ground flat and has a diameter of about 7 mm. When full it contains a constant number (about 56) of drops of distilled water at 15° C., and all the drops, which can be counted as they fall from the ground base, are of equal size. Fractions can also be estimated. Ascoli first applied this principle to typhoid sera. The diluted serum was mixed with a fixed quantity of alcoholic extract of B. typhosus and the number of drops immediately counted. The preparation was then incubated for two hours, cooled, and the drops again counted. With very dilute antigens there was an increase of two or three drops, but no increase was obtained with normal sera nor with an antigen prepared from B. coli. Similar results were obtained in syphilis, and the reaction was then extended to cancer. The main difficulty in this test as applied to cancer is the preparation of the antigen, which appears to be a lipoid body. Several methods have been adopted from time to time, and it has been shown that only a certain proportion of cancerous tumours are suitable for preparing it, and it is not very stable. The most recent antigen is a methyl alcohol extract, which is titrated against normal serum, the weakest dilution which fails to give an increase of more than one drop with diluted normal serum being used. Stronger dilutions of the antigen are unreliable, because they give a positive reaction with normal sera.

As to the specific character of this reaction, it has been shown that a positive result can be obtained occasionally in other diseases, such as diabetes, tubercle, febrile and septic conditions, but it is not a cachexia reaction. This appears to be the most satisfactory of all the laboratory tests ; the main difficulty consists in obtaining a satisfactory antigen. Ascoli and Izar obtained a positive reaction in over 93 per cent. of cancer cases, Verson in 55 per cent., Stabilini in 100 per cent., Kelling in 47 per cent., Michaeli and Cattoretti in 87 per cent., and Stammler in 73 per cent. The last named also obtained a positive reaction in 20 per cent. of non-malignant cases. Tadesco obtained 93 per cent. positive in malignant disease and 12 per cent. positive or doubtful in other cases. Kraus and others got 92 per cent. positive in malignant cases.

Stammler has modified this test, and instead of using the drop reaction, relies on the formation of a precipitate when the antigen is incubated with the patient's serum. He obtained 83 per cent. positive results with cancer cases and 14 per cent. with other diseases.

### III. Tests applied to the Urine.

I now pass on to consider those tests which are applied to the urine. These are all devised with a view to showing either that certain changes of metabolism have been set up by the cancerous process which can be recognised by the chemical character of the excretions, or that certain ferments are being excreted as a concomitant of the cancerous condition, which are not present to the same extent in health.

1. The first of the latter group is the presence of a reducing ferment in the urine capable of decolourising methylene blue.[18] This test has the advantage of extreme simplicity, for all that is necessary is to mix the urine with the ordinary solution of methylene blue and leave it to stand in a warm place for some hours. If the colour is destroyed a reductase is present and the test is positive. The disadvantage of this test is that it has been shown to be quite unreliable, as all cancer cases do not give a positive result and many other conditions do.

2. More complicated is the determination of the proteolytic ferment in the urine.[19] The method used consists of incubating the clear urine in various concentrations with a dilute solution of ricin, which has been precipitated with decinormal hydrochloric acid. However, the published results are contradictory, for two reasons—firstly, that different experimenters have adopted different methods ; and secondly, that in many cases the clinical diagnosis has not been confirmed. In cases of simple achylia urinary pepsin may be low or high as compared with gastric pepsin, and in some cases of cancer it is present in large amounts in the urine. On the other hand, it may be absent completely in achylia at certain times. Some cancers also show complete absence of urinary pepsin. Thus, though pepsin in large amounts in the urine may point to cancer, the results on the whole are too variable to be of clinical value, and it is especially in early cases that these variable results are obtained.

3. A large amount of work has recently been done on the urinary nitrogenous bodies in relation to cancer. It seems clear that under certain abnormal metabolic conditions there are variations in the form in which nitrogen is excreted, but so far the results do not seem sufficiently definite to be of great value.

The principal methods which have been suggested are—

(*a*) Precipitation with 5 per cent. phosphomolybdic acid. The urobilin must be removed by washing the precipitate with absolute alcohol. The occurrence of a precipitate is only suggestive of cancer, and only occurs in about half the cases.[20]

(*b*) Increase in the proportion of nitrogenous substances precipitated by alcohol. This is known as Salkowski's test, or the colloidal nitrogen test.[21] An increase in the proportion of colloidal nitrogen was considered diagnostic of cancer. Later, instead of alcohol, salts of the heavy metals, such as subacetate of lead or zinc chloride, were used as precipitants after removing the albumin with baryta mixture. Lower values are obtained by this method.

Confirmatory evidence of the value of these methods is wanting, the conclusions of other authors being summarised as follows :—

(i) Higher values of colloidal nitrogen are not specific for cancer. Not only do some cases give a low percentage of colloidal nitrogen, but there is a large series of cases, especially cirrhosis of the liver, diabetes, and infectious fevers, which give more or less constantly high figures.

(ii) With these exceptions it is true that cancer cases on an average give a higher percentage than healthy persons or non-cancerous cases.

(iii) The condition of nutrition, the food and the nitrogen balance influence the result of the test.

(*c*) A similar method is the estimation of the polypeptids in the urine.[22] The amino-acids are first estimated by formalin titration ; the polypeptids are then split up by means of strong HCl, the acid removed, and the resulting amino-acids again estimated. The difference gives the amount of polypeptids. Although polypeptids on an average are increased in cancer, yet this method is open to the same criticism as the last.

(*d*) A peculiar group of polypeptids, namely the oxyproteinic acids,[23] have also been found to be increased in cancer. The method for their estimation by Solomon and Saxl depends on their sulphur content. The method is very elaborate, as the inorganic sulphur and ethereal sulphates have first to be removed, and the polypeptid sulphur then oxidised by perhydrol or sodium nitrite.

This reaction has now been applied to about 223 cases, with positive results in about 70 per cent. It does not appear to be due to cachexia ; it also occurs in other conditions (5 per cent.), but it seems to appear early in cancer cases. The elaborate technique is against its general adoption.

4. A reaction differing somewhat from those in the last group was described in 1910 by Royle.[24] She found that in cancer patients the ratio of the urinary phosphates to the uric acid was decreased, that is to say, that on a standard diet the excretion of phosphates decreased and the excretion of uric acid increased. As yet the results have not been confirmed on a large scale, but the reaction appears to be so similar to those previously described that it seems probable that it will be shown to be dependent on some condition frequent in cancer, but common to it and certain other disordered states of metabolism.

The examination of the urine cannot be said to have hitherto provided a satisfactory aid to the diagnosis of cancer. The methods are for the most part complicated, and the tests have in no case been shown to be pathognomonic. They appear to occur early in the disease, however, and to be dependent on metabolic changes other than mere cell destruction or cachexia.

As far as the results hitherto obtained go, a positive result from the meiostagmin reaction and the method of Solomon and Saxl for estimating the oxyproteinic sulphur would seem to give a very strong presumption in favour of cancer, provided that certain other conditions can be otherwise excluded. On the negative side, an absence of increase in the antitryptic power of the serum and an increase in the lipoclastic accelerators is against the presence of malignant disease.

### BIBLIOGRAPHY.

[1] Moore, Roaf and Kelly, *Biochem. J.*, 1906, i. 274, 297.
Idem, *Brit. M. J.*, 1906, ii. 213.
Boas, *Deutsche med. Wchnschr.*, 1911, No. 49.

[2] v. Aldor, *Wien. klin. Wchnschr.*, 1907, xx. 598.
A. J. Clark, *St. Barth. Hosp. Rep.*, 1910, xlv. 97.
Volkmann's *Sammlung*, Neue Folge, No. 387, 1906.

[3] Sigel, *Berl. klin. Wchnschr.*, 1904, xli. 299, 338.
Salomon, *Deutsche med. Wchnschr.*, 1903, No. 31.
Mathieu, *J. de Méd. et de Chir. prat.*, Sept. 10th, 1909.

[4] Mansell Moullin, *Lancet*, 1910, i. 417.

[5] Sigel, *loc. cit.*
Gluzinski, *Wien. Klin. Wchnschr.*, 1912, xxv. 553.
Alfredo, *Riforma Med.*, March 24th, 1908.

[6] Wolff and Junghans, *Berl. klin. Wchnschr.*, 1911, xlviii. 978.
Thiele, *Ibid.*, 1912, xlix. 544.
Ernstein, *Med. klin.*, March 24th, 1912, p. 484.

[7] Kuttner and Pulvermacher, *Berl. klin. Wchnschr.*, 1910, xlvii. 2057.
Ehrenberg, *Ibid.*, 1911, xlviii. 704.
Sigel and Alfredo, *loc. cit.*
Neubauer and Fischer, *Deutsche Arch. f. Klin. Med.*, 1909, xcvii. 499.
Lyle and Kober, *N. York M. J.*, 1910, xci. 1151.
Weinstein, *J. Am. M. Ass.*, 1910, lv. 1085.

[8] Schryver and Singer, *Quart. J. Med.*, 1912, vi. 71.

[9] Liveriato, *Centralbl. f. Bakteriol.*, 1910, lv. 510.
Pfeiffer and Finsterer, *Wien. klin. Wchnschr.*, 1909, xxii. 989.

[10] Alfredo, *loc. cit.*

[11] Macalister and Ross, *Proc. Roy. Soc. Med.* (Med. Sect.), 1908, ii. 50.

[12] Marcus, *Berl. klin. Wchnschr.*, 1908, xlv. 689.
Brieger and Trebing, *Ibid.*, p. 1041, 1349, 2260.
Hort, *Brit. M. J.*, 1909, ii. 966.

[13] Shaw-Mackenzie and Rosenheim, *Proc. Roy. Soc. Med.*, 1910, iii. (Pathol. Sect.) 168.
Shaw-Mackenzie, *Ibid.*, 1912, v. (Therap. and Pharmacol. Sect.), 152, 1912.

[14] Kraus, v. Graff and Ransi, *Wien. klin. Wchnschr.*, 1911, xxiv. No. 28.
Monakow, *München. med. Wchnschr.*, 1911, lviii. 2207.

[15] Elsberg, *J. Am. M. Ass.*, 1909, lii. 1036.
Elsberg, Neuhof and Geist, *Am. J. M. Sc.*, 1910, cxxxix. 264.
Crile, *J. Am. M. Ass.*, 1908, l. 1583, li. 2036, 1908.
Warfield, *Arch. of Int. Med., Chicago*, No. 15, 1911.
Simon, *J. Am. M. Ass.*, 1908, li. 915.
Gorham and Lisser, *Am. J. M. Sc.*, 1912, cxliv. 103.
See also *Brit. M. J.*, 1912, i. 1138 (references).

16 *Brit. M. J.*, 1912, i. 1030 (references).
17 Traube, *Pflüger's Archiv.*, 1908, No. 123, p. 419.
Ascoli, *München. med. Wchnschr.*, 1910, lvii. 62.
Ascoli and Izar, *Ibid.*, p. 403.
Bernstein and Simons, *Am. J. M. Sc.*, 1911, cxlii. 852 (references).
*Brit. M. J.*, 1912, i. 1081 (references).
Leidi, *Berl. klin. Wchnschr.*, 1911, xlviii. 1716.
Verson, *Wien. klin. Wchnschr.*, 1910, xxiii. 1102.
Stabilini, *Berl. klin. Wchnschr.*, 1910, xlvii. 1498.
Kelling, *Wien. klin. Wchnschr.*, 1911, xxiv. No. 3.
Michaeli and Cattoretti, *Ibid.*, 1910, xxiii. 1555.
Stammler, *Munchen. med. Wchnschr.*, 1911, lviii. 1957.
Idem, *Arch. f. klin. Chir.*, 1911, xcvi. 158.
Tadesco, *Wien. klin. Wchnschr.*, 1909, xxii. 954.
Izar, *Ibid.*, 1912, No. 33.
18 Fuhs and Lintz, *J. Am. M. Ass.*, 1911, lvi. 1882.
Verbrycke, *Med. Rec.*, 1911, lxxx. 876.
19 Bieling, *Deutsche Arch. f. klin. Med.*, 1911, cii. 507.
20 Ascoli and Grazia, *Berl. klin. Wchnschr.*, 1901, xxxviii. 1009 (references).
Ury and Lilienthal, *Arch. f. Verdauungskr.*, 1905. xi.
Von Aldor, *loc. cit.*
21 Salkowski, *Berl. klin. Wchnschr.*, 1905, xlii. 1581.
Idem, *Ibid.*, 1910, xlvii. 533, 1746, 2297.
Mancini, *Deutsche Arch. f. klin. Med.*, 1911, ciii. 288.
Ischioka, *Med. Klin.*, 1911, vii. 1548.
Gross and Reh, *Ibid.*, 1911, vii. 778.
Kojo, *Ztschr. f. Physiol. Chem.*, 1911, lxxiii. 416.
22 Sörensen, *Biochem. Ztschr.*, Bd. vii.
Frey and Gigon, *Ibid.*, Heft 304.
Henriques, *Ztschr. f. physiol. chem.*, Bd. lx.
Falk, Solomon and Saxl, *Med. Klin.*, 1910, vi. 510.
23 Abderhalden and Pregl, *Ztschr. f. Physiol. Chem.*, Bd. xlvi., 1905.
Solomon and Saxl, *Wien. klin. Wchnschr.*, 1911, xxiv. 449.
Kaldeck, *Ibid.*, p. 1681.
Mazzitelli, *Riforma Med.*, July 27th, 1912.
24 Royle, *Lancet*, 1910, ii. 449.

